# Humoral Responses to Single-Dose BNT162b2 mRNA Vaccination in Dialysis Patients Previously Infected With SARS-CoV-2

**DOI:** 10.3389/fmed.2021.721286

**Published:** 2021-08-17

**Authors:** Claudius Speer, Christian Morath, Maximilian Töllner, Mirabel Buylaert, Daniel Göth, Christian Nusshag, Florian Kälble, Matthias Schaier, Julia Grenz, Martin Kreysing, Paula Reichel, Asa Hidmark, Gerald Ponath, Paul Schnitzler, Martin Zeier, Caner Süsal, Katrin Klein, Louise Benning

**Affiliations:** ^1^Department of Nephrology, University of Heidelberg, Heidelberg, Germany; ^2^Molecular Medicine Partnership Unit Heidelberg, European Molecular Biology Laboratory (EMBL), Heidelberg, Germany; ^3^Department of Gastroenterology and Hepatology, University of Heidelberg, Heidelberg, Germany; ^4^Virology, Department of Infectious Diseases, University of Heidelberg, Heidelberg, Germany; ^5^Department of Transplantation Immunology, Institute of Immunology, University of Heidelberg, Heidelberg, Germany

**Keywords:** SARS-CoV-2, COVID-19, hemodialysis, immune response, vaccination

## Abstract

Seroconversion rates following infection and vaccination are lower in dialysis patients compared to healthy controls. There is an urgent need for the characterization of humoral responses and success of a single-dose SARS-CoV-2 vaccination in previously infected dialysis patients. We performed a dual-center cohort study comparing three different groups: 25 unvaccinated hemodialysis patients after PCR-confirmed COVID-19 (Group 1), 43 hemodialysis patients after two-time BNT162b2 vaccination without prior SARS-CoV-2 infection (Group 2), and 13 single-dose vaccinated hemodialysis patients with prior SARS-CoV-2 infection (Group 3). Group 3 consists of seven patients from Group 1 and 6 additional patients with sera only available after single-dose vaccination. Anti-S1 IgG, neutralizing antibodies, and antibodies against various SARS-CoV-2 protein epitopes were measured 3 weeks after the first and 3 weeks after the second vaccination in patients without prior SARS-CoV-2 infection, 6 weeks after the onset of COVID-19 in unvaccinated patients, and 3 weeks after single-dose vaccination in patients with prior SARS-CoV-2 infection, respectively. Unvaccinated patients after COVID-19 showed a significantly higher neutralizing antibody capacity than two-time vaccinated patients without prior COVID-19 [median (IQR) percent inhibition 88.0 (71.5–95.5) vs. 50.7 (26.4–81.0); *P* = 0.018]. After one single vaccine dose, previously infected individuals generated 15- to 34-fold higher levels of anti-S1 IgG than age- and dialysis vintage-matched unvaccinated patients after infection or two-time vaccinated patients without prior SARS-CoV-2 infection with a median (IQR) index of 274 (151–791) compared to 18 (8–41) and 8 (1–21) (for both *P* < 0.001). With a median (IQR) percent inhibition of 97.6 (97.2–98.9), the neutralizing capacity of SARS-CoV-2 antibodies was significantly higher in single-dose vaccinated patients with prior SARS-CoV-2 infection compared to other groups (for both *P* < 0.01). Bead-based analysis showed high antibody reactivity against various SARS-CoV-2 spike protein epitopes after single-dose vaccination in previously infected patients. In conclusion, single-dose vaccination in previously infected dialysis patients induced a strong and broad antibody reactivity against various SARS-CoV-2 spike protein epitopes with high neutralizing capacity.

## Introduction

Patients on maintenance hemodialysis are at great risk for severe courses of coronavirus disease 2019 (COVID-19) caused by severe acute respiratory syndrome coronavirus type 2 (SARS-CoV-2) (1). An urgent need has been issued to prioritize this vulnerable cohort in international vaccine programs and to determine vaccination response (2).

Recently, we and others demonstrated a lower humoral response to BNT162b2 mRNA vaccine in dialysis patients compared to healthy controls, with particularly low seroconversion rates after the first vaccine dose (3, 4). After COVID-19 vaccination and infection, neutralizing anti-receptor-binding domain antibodies increase during the first 2 months and decline subsequently over time (5, 6). However, little is known about differences in the humoral response of dialysis patients after COVID-19 disease and SARS-CoV-2 vaccination.

Rapid population vaccination coverage is being sought, especially in countries with shortage of vaccine and high COVID-19 incidence. Humoral and cellular responses following COVID-19 disease may make alternative vaccination strategies necessary for previously infected individuals (7, 8). Single-dose rather than double-dose administration might be a reasonable vaccination strategy for individuals recovered from prior infection. First studies showed a strong anti-spike antibody response including neutralizing antibodies in healthy individuals following COVID-19 disease after only a single-dose of mRNA vaccine (9–12). However, since sensitivity to the BNT162b2 mRNA vaccine is lower in patients on dialysis with limited seroconversion rates after only one vaccine dose, there is an urgent need to determine the success of single-dose vaccination in previously infected patients. This is one of the first studies providing in-depth characterization of humoral responses following single-dose vaccination in dialysis patients with confirmed prior SARS-CoV-2 infection.

## Materials and Methods

### Study Design

**Figure 1:**
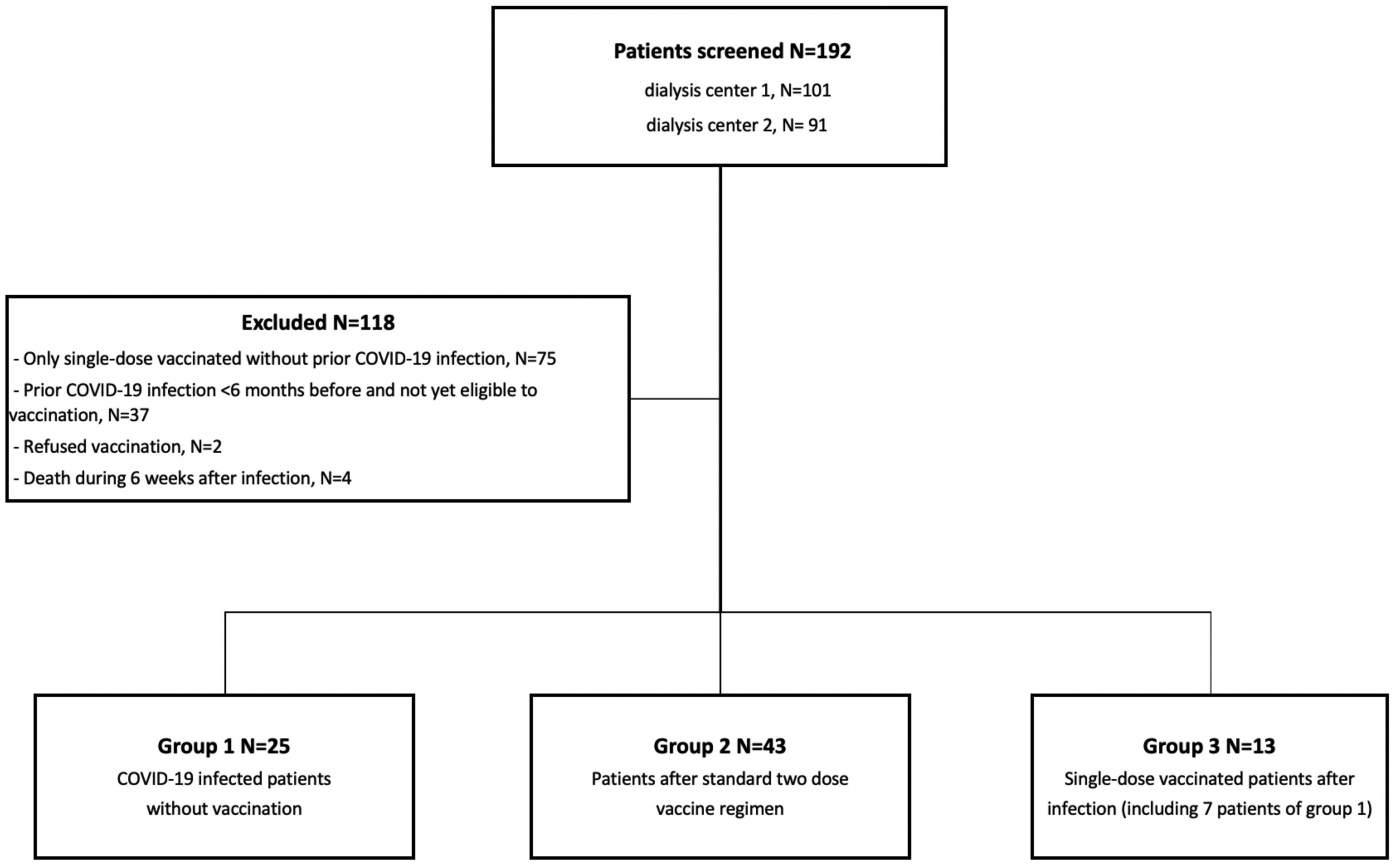
Flowchart of study population selection including screening, exclusion, and patients eligible for study participation. One hundred and ninety-two patients were screened for study participation at two sites. One hundred and eighteen individuals were excluded, and the remaining 74 individuals were found to be eligible for study participation. Depending on COVID-19 infection or vaccination status, individuals were separated into three different groups, namely COVID-19 infected individuals, patients who received a full BNT162b2 vaccination, and previously COVID-19 infected individuals who received a single-dose vaccination. *N* indicates the number of individuals. ^a^Group 3 contains seven patients of group 1 after single-dose BNT162b2 vaccination.

In this dual-center observational cohort study, we screened 192 patients on dialysis between March 2020 and April 2021 at the Division of Nephrology of the University Hospital of Heidelberg and at the associated Kidney Center Heidelberg for inclusion (Figure 1). Eligible participants were either hemodialysis patients after PCR-confirmed COVID-19 (Group 1), uninfected hemodialysis patients after two-time mRNA BNT162b2 (BioNTech) vaccination (Group 2), or previously COVID-19 infected individuals who received a single-dose of BNT162b2 ≥ 6 months after infection (Group 3) (Figure 1). Group 3 consists of seven patients from Group 1 and 6 patients without available sera who are not part of Group 1. We exclusively included patients on hemodialysis.

The anti-S1 IgG index, SARS-CoV-2 specific neutralizing antibodies, and a bead-based differentiation of SARS-CoV-2 target antibodies were measured in 43 BNT162b2 vaccinated dialysis patients without prior SARS-CoV-2 infection 3 weeks after the first and 3 weeks after the second vaccination. In 25 unvaccinated dialysis patients after COVID-19, humoral responses were measured 6 weeks after the onset of infection. The onset of infection was defined as the day with the first symptoms and the diagnosis was made by positive PCR. Vaccination was performed twice in all 43 patients of Group 1 at week 0 and 3. To exclude patients with prior SARS-CoV-2 infection or infection during follow-up, we measured antibodies to the nucleocapsid protein before enrollment and after the first and the second vaccination. SARS-CoV-2 infected dialysis patients who died within the first 6 weeks after disease onset were excluded. In addition, 13 dialysis patients received a single-dose of BNT162b2 vaccine at least 6 months after onset of COVID-19 disease and humoral responses were subsequently measured 3 weeks after single-dose vaccination. To exclude a decisive effect of age and dialysis vintage on humoral responses, we performed a subgroup analysis with age-matched and dialysis vintage-matched COVID-19 infected (*N* = 20), fully vaccinated (*N* = 30), and previously infected single-dose vaccinated (*N* = 13) dialysis patients, respectively (Supplementary Tables 1–3). To detect breakthrough infections after vaccination or previous infection, we performed rapid SARS-CoV-2 antigen testing before each individual dialysis session (three times weekly) and PCR testing once a week.

The study (ethics number: S-061/2021) was approved by the ethics committee of the University of Heidelberg on 02/15/2021 and conducted in accordance with the Declaration of Helsinki. Written informed consent was obtained from all study participants.

### Anti-SARS-CoV-2 IgG Assay

The IgG response against the S1 protein was measured by the SARS-CoV-2 IgG Assay (Siemens, Eschborn, Germany) and the total antibody response against the nucleocapsid protein by the Elecsys Anti-SARS-CoV-2 assay (Roche, Mannheim, Germany) according to the manufacturer's instructions. A semi-quantitative index of <1 was classified as negative, and a value of ≥1 or higher as positive. This cutoff for detection gives a specificity of 99.9% with a sensitivity of 96.4% for the Siemens spike assay.

### Neutralizing Capacity of SARS-CoV-2 Antibodies

The binding-inhibition potency of serum samples was detected by a plate-based SARS-CoV-2 surrogate virus neutralizing assay (Medac, Wedel, Germany) (13, 14). This assay mimics the virus-host interaction by direct protein-protein interaction using purified receptor-binding domain protein from the viral spike protein and the host cell receptor angiotensin converting enzyme 2 (ACE2). The antibodies in serum samples were incubated with SARS-CoV-2 receptor-binding domain horse-radish peroxidase and added to ACE2 coated wells. The reactions were developed using 3,3′,5,5′-tetramethylbenzidine as substrate. Optical density at 450 nm was measured in each well and the percent (%) inhibition was calculated as presented in the Supplementary Material. A cut-off for viral neutralization of ≥30% inhibition of receptor-binding domain:ACE-2 binding was applied according to the manufacturer's instructions, resulting in a specificity of 100% with a sensitivity of 98%. The SARS-CoV-2 anti-S1 IgG index (Siemens, Eschborn, Germany) correlated with the neutralizing antibody activity measured by the surrogate virus neutralization assay in our cohort (Supplementary Figure 1).

### Bead-Based Multiplex Assay for SARS-CoV-2 Antibody Detection

We determined IgG antibodies against different SARS-CoV-2 target antigens by a multiplex bead-based assay (One Lambda Inc., West Hill, CA) for the Luminex platform (LabScreen COVID Plus), including besides the SARS-CoV-2 nucleocapsid protein, four distinct fragments of the SARS-CoV-2 spike protein, namely the full spike protein, the S1 protein, the receptor-binding domain of the spike protein, and the S2 protein (15). Additionally, we measured S1 fragments from six other coronaviruses, namely HCoV-229E, HCoV-HKU1, HCoV-NL63, HCoV-OC43, MERS-CoV, and SARS-CoV-1. Antibody detection was performed according to the manufacturer's instructions and the mean fluorescence intensity (MFI) was analyzed on a Luminex 200 device (Luminex Corporation, Noord-Brabant, The Netherlands). The MFI cutoff values for each of the 11 proteins are shown in Supplementary Table 4.

### Monitoring of Adverse Events

Adverse events assessment included medical monitoring of local and systemic adverse events for 10 days after the first and the second vaccine dose at each in-house dialysis treatment. Adverse events were assessed using a 12-item questionnaire inquiring previously mentioned side-effects and the use of pain medication after vaccine reception. The questionnaire is presented in the Supplementary Material. Patients were further screened for infection with COVID-19 three times a week by rapid antigen testing and once a week by PCR testing.

### Statistics

Data are expressed as median and interquartile range (IQR). Results of different groups were compared by applying the Kruskal-Wallis test with Dunn's post-test. Statistical analysis of categorical data was performed using the chi-square (χ^2^) test. Statistical significance was assumed at a *P* < 0.05. The statistical analysis was performed using GraphPad Prism version 9.0.0 (GraphPad Software, San Diego CA, USA).

## Results

### Baseline Characteristics and Courses of COVID-19 Disease

Twenty-five patients had a PCR-confirmed COVID-19 disease, 43 patients received the standard two-dose regimen of BNT162b2 mRNA vaccine, and 13 patients received a single-dose BNT162b2 vaccination at least 6 months after the onset of COVID-19 disease (Figure 1). Baseline characteristics of the different groups are shown in Table 1. We included only patients on hemodialysis in our study. COVID-19 infected patients were significantly younger compared to fully vaccinated or previously infected dialysis patients vaccinated with a single-dose (median age 74 vs. 82 vs. 79 years; *P* = 0.002). An age- and dialysis vintage-adjusted subgroup analysis was performed and baseline characteristics as well as COVID-19 related clinical characteristics are shown in Supplementary Tables 1, 2, respectively. Antibody responses were determined 6 weeks after the onset of COVID-19 disease, 3 weeks after the first and 3 weeks after the second vaccine dose, or 3 weeks after single-dose vaccination in patients with onset of COVID-19 disease in median (IQR) 6.3 (6.1–6.7) months before.

**Table 1:** Baseline characteristics of participating dialysis patients.

	**Group 1**	**Group 2**	**Group 3^**a**^**	***P-*value**
	**(COVID-19 infected)**	**(Twice BNT162b2 vaccinated)**	**(Single-dose BNT162b2 vaccinated after infection)**	
Number of patients, *N*	25	43	13	
Age at enrollment (years), median (IQR)	74 (49–82)	82 (80–87)	79 (73–84)	0.002^b^
Gender (female), N (%)	8 (32)	16 (37)	2 (15)	0.34
BMI, median (IQR)	26 (23–29)	25 (22–28)	25 (23–27)	0.39
Dialysis vintage (months), median (IQR)	25 (12–64)	44 (22–84)	41 (19–78)	0.24
Cause of nephropathy				
Diabetes, *N* (%)	5 (20)	10 (23)	2 (15)	0.82
Vascular, *N* (%)	6 (24)	8 (19)	3 (23)	0.85
Polycystic kidney disease, *N* (%)	1 (4)	3 (7)	0 (0)	0.58
Glomerulonephritis, *N* (%)	5 (20)	11 (26)	4 (31)	0.75
Chronic pyelonephritis, *N* (%)	1 (4)	1 (2)	0 (0)	0.75
Other, *N* (%)	7 (28)	10 (23)	4 (31)	0.83
Comorbidities				
Arterial hypertension, *N* (%)	20 (80)	43 (100)	10 (77)	0.006^b^
Diabetes, *N* (%)	11 (44)	18 (42)	5 (39)	0.95
Cancer, *N* (%)	6 (24)	9 (21)	3 (23)	0.96
Immunosuppressants, *N* (%)	6 (24)	8 (19)	3 (23)	0.85
Previous transplant, *N* (%)	3 (12)	5 (12)	1 (8)	0.92
Smoker (active and former), *N* (%)	8 (32)	15 (35)	5 (39)	0.92
CAD, *N* (%)	16 (64)	28 (65)	7 (71)	0.93
PAD, *N* (%)	5 (20)	15 (35)	4 (31)	0.43
Chronic lung disease, *N* (%)	7 (28)	12 (28)	4 (31)	0.98
Chronic liver disease, N (%)	5 (20)	4 (9)	2 (15)	0.45

*BMI, body mass index; CAD, coronary artery disease; PAD, peripheral artery disease.*

a *Group 3 contains seven patients of Group 1 after single-dose BNT162b2 vaccination and six patients without available sera after infection who are not part of Group 1.*

b *Statistically significant, results of different groups were compared by applying the Kruskal-Wallis test with Dunn's post-test*.

The clinical courses of individuals infected with SARS-CoV-2 are shown in Table 2. According to previously used grading systems for COVID-19 disease severity (16, 17), three patients had critical (12%), three severe (12%), fourteen moderate (56%), and five mild (20%) COVID-19 disease courses (Table 2; Supplementary Table 4). No individual was asymptomatic. Dialysis patients with critical COVID-19 disease courses were more often male and suffered more frequently from diabetes (for both *P* < 0.05; Table 2). Four patients died within 6 weeks after onset of COVID-19 disease and were subsequently excluded (Figure 1).

**Table 2:** Clinical characteristics and COVID-19 disease courses of dialysis patients with COVID-19 infection.

**COVID-19 disease severity**	**Mild^**b**^**	**Moderate**	**Severe**	**Critical**	**All patients**	***P-*value**
Number of patients, *N* (%)	5 (20)	14 (56)	3 (12)	3 (12)	25 (100)	
Age at enrollment (years), median (IQR)	49 (43–85)	76 (57–82)	75 (73–91)	61 (46–77)	74 (49–82)	0.73
Gender (female), *N* (%)	4 (80)	2 (14)	2 (67)	0 (0)	8 (32)	0.015^a^
BMI, median (IQR)	27 (24–29)	26 (23–30)	29 (26–30)	27 (23–29)	26 (23–29)	0.84
Dialysis vintage (months), median (IQR)	36 (10–111)	25 (8–69)	23 (19–72)	34 (10–35)	25 (12–64)	0.99
Cause of nephropathy						
Diabetes, *N* (%)	0 (0)	3 (21)	1 (33)	1 (33)	5 (20)	0.69
Vascular, *N* (%)	1 (20)	3 (21)	1 (33)	1 (33)	6 (24)	0.84
Polycystic kidney disease, *N* (%)	1 (20)	0 (0)	0 (0)	0 (0)	1 (4)	0.71
Glomerulonephritis, *N* (%)	2 (40)	2 (14)	0 (0)	1 (33)	5 (20)	0.28
Chronic pyelonephritis, *N* (%)	1 (20)	0 (0)	0 (0)	0 (0)	1 (4)	0.45
Other, *N* (%)	0 (0)	6 (43)	1 (33)	0 (0)	7 (28)	0.17
Comorbidities						
Arterial hypertension, *N* (%)	4 (80)	11 (79)	3 (100)	2 (67)	20 (80)	0.82
Diabetes, *N* (%)	0 (0)	6 (43)	2 (67)	3 (100)	11 (44)	0.034^a^
Cancer, *N* (%)	0 (0)	4 (29)	1 (33)	1 (33)	6 (24)	0.61
Immunosuppressants, *N* (%)	1 (20)	2 (14)	1 (33)	2 (67)	6 (24)	0.29
Previous transplant, *N* (%)	1 (20)	1 (7)	0 (0)	1 (33)	3 (12)	0.47
Smoker (active and former), *N* (%)	1 (20)	5 (36)	1 (33)	1 (33)	8 (32)	0.86
CAD, *N* (%)	3 (60)	9 (64)	1 (33)	3 (100)	16 (64)	0.41
PAD, *N* (%)	1 (20)	2 (14)	0 (0)	2 (67)	5 (20)	0.20
Chronic lung disease, *N* (%)	2 (40)	3 (21)	1 (33)	1 (33)	7 (28)	0.83
Chronic liver disease, *N* (%)	1 (20)	3 (21)	0 (0)	1 (33)	5 (20)	0.86
Initial symptoms						
Fever, *N* (%)	3 (60)	12 (86)	1 (33)	3 (100)	19 (76)	0.31
Cough, *N* (%)	1 (20)	7 (50)	1 (33)	2 (67)	11 (44)	0.54
Headache, *N* (%)	0 (0)	1 (7)	0 (0)	1 (33)	2 (8)	0.75
Diarrhea, *N* (%)	1 (20)	1 (7)	0 (0)	1 (33)	3 (12)	0.81
Fatigue, *N* (%)	3 (60)	7 (50)	0 (0)	2 (33)	12 (48)	0.38
Dyspnoe, *N* (%)	0 (0)	4 (29)	3 (100)	2 (33)	9 (36)	0.31
Oxygen supply, *N* (%)	0 (0)	4 (29)	3 (100)	3 (100)	10 (40)	0.004^a^
Non-invasive ventilation, *N* (%)	0 (0)	0 (0)	3 (100)	3 (100)	6 (24)	0.001^a^
Invasive ventilation, *N* (%)	0 (0)	0 (0)	0 (0)	3 (100)	3 (12)	0.001^a^
Use of vasopressors, *N* (%)	0 (0)	0 (0)	0 (0)	3 (100)	3 (12)	0.001^a^
Days of ICU stay, median (IQR)	0	0	4 (2–5)	49 (43–56)		0.001^a^
Days on ventilation, median (IQR)	0	0	2 (0–5)	36 (23–37)		0.001^a^
Immunomodulatory therapy						
Dexamethasone, *N* (%)	0 (0)	1 (7)	2 (67)	3 (100)	6 (24)	0.001^a^
Plasma exchange, *N* (%)	0 (0)	0 (0)	1 (33)	2 (67)	3 (12)	0.006^a^
Days of PCR positivity, median (IQR)	22 (17–39)	26 (12–35)	34 (18–34)	43 (29–43)	29 (16–39)	0.32

*BMI, body mass index; CAD, coronary artery disease; PAD, peripheral artery disease; PCR, polymerase chain reaction; ICU, intensive care unit.*

a *Statistically significant, results of different groups were compared by applying the Kruskal-Wallis test with Dunn's post-test.*

b *Previously used grading systems for COVID-19 disease severity (16, 17)*.

We did not detect symptomatic or asymptomatic breakthrough infections in any of the three different groups during follow-up.

### Antibody Responses in Dialysis Patients After COVID-19 Disease or Two Times BNT162b2 mRNA Vaccination

**Figure 2:**
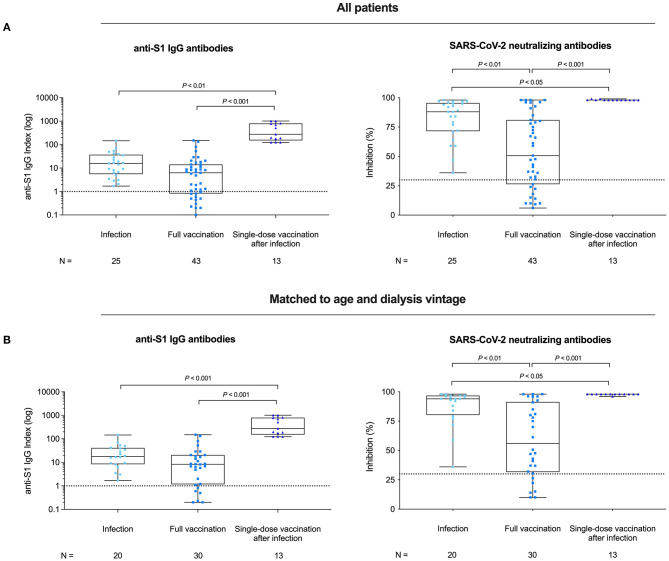
Spike antigen-specific SARS-CoV-2 IgG levels and SARS-CoV-2 neutralizing capacity in hemodialysis patients after COVID-19 infection, complete BNT162b2 vaccination, and in previously infected patients receiving a single dose of BNT162b2. SARS-CoV-2 IgG antibodies and SARS-CoV-2 neutralizing capacity of vaccine-induced antibodies in all **(A)** and in age- and dialysis vintage-adjusted patients **(B)** for COVID-19 infected patients, patients receiving complete BNT162b2 vaccination and for previously COVID-19 infected patients receiving a single dose of BNT162b2, respectively. SARS-CoV-2 IgG antibodies are represented logarithmically as an anti-S1 IgG index. The dashed line represents the cutoff for SARS-CoV-2 IgG antibody detection with a semi-quantitative index of <1 classified as negative, and a value of β ≥ 1 or higher classified as positive. SARS-CoV-2 neutralizing capacity of BNT162b2 induced antibodies was determined by a virus neutralization test and antibody-mediated inhibition of the SARS-CoV-2 receptor-binding domain:angiotensin-converting enzyme 2 interaction is expressed as a percentage. Binding inhibition >30% indicates presence of SARS-CoV-2 neutralizing antibodies above the limit of detection of this test.

Dialysis patients after SARS-CoV-2 infection tended to have a higher anti-S1 IgG level as compared to dialysis patients after two times BNT162b2 vaccination (median index 15.8 vs. 6.3; IQR 5.6–37.1 vs. 0.8–14.2; *P* = 0.060; Figure 2A). Whereas, 25/25 (100%) of SARS-CoV-2 infected dialysis patients had antibody levels above the index cutoff for detection, only 31/43 (72%) of twice vaccinated patients exceeded this threshold.

We also determined the SARS-CoV-2 neutralizing capacity in both groups. Sera of infected patients showed a significantly higher inhibition than sera of twice vaccinated dialysis patients (median of percent inhibition 88.0 vs. 50.7; IQR 71.5–95.5 vs. 26.4–81.0; *P* = 0.018; Figure 2A). An age- and dialysis vintage-adjusted subgroup analysis revealed similar results for neutralizing antibody capacity (*P* = 0.047; Figure 2B; Supplementary Table 3).

**Figure 3:**
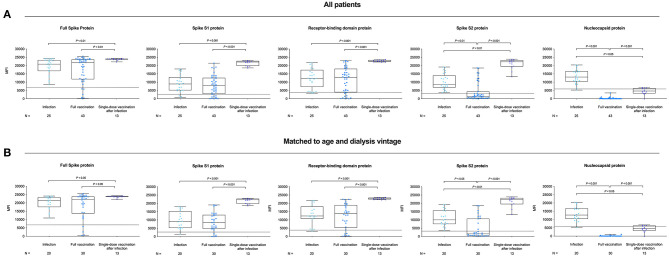
IgG antibodies against different SARS-CoV-2 target antigens in hemodialysis patients after COVID-19 infection, complete BNT162b2 vaccination, and in previously infected patients receiving a single dose of BNT162b2. Bead-based detection of antibodies against the full spike, the S1 spike, the receptor-binding domain of the spike, the S2 spike, and the nucleocapsid protein of SARS-CoV-2 in all dialysis patients of different groups **(A)** and in an age- and dialysis vintage-matched subgroup analysis **(B)**. The x-axis represents the sample number, the y-axis the mean fluorescence intensity (MFI), and the dashed line indicates the cutoff for each SARS-CoV-2 target antigen in all patients **(A)** and in matched to age and matched to dialysis vintage patients **(B)**.

**Figure 4:**
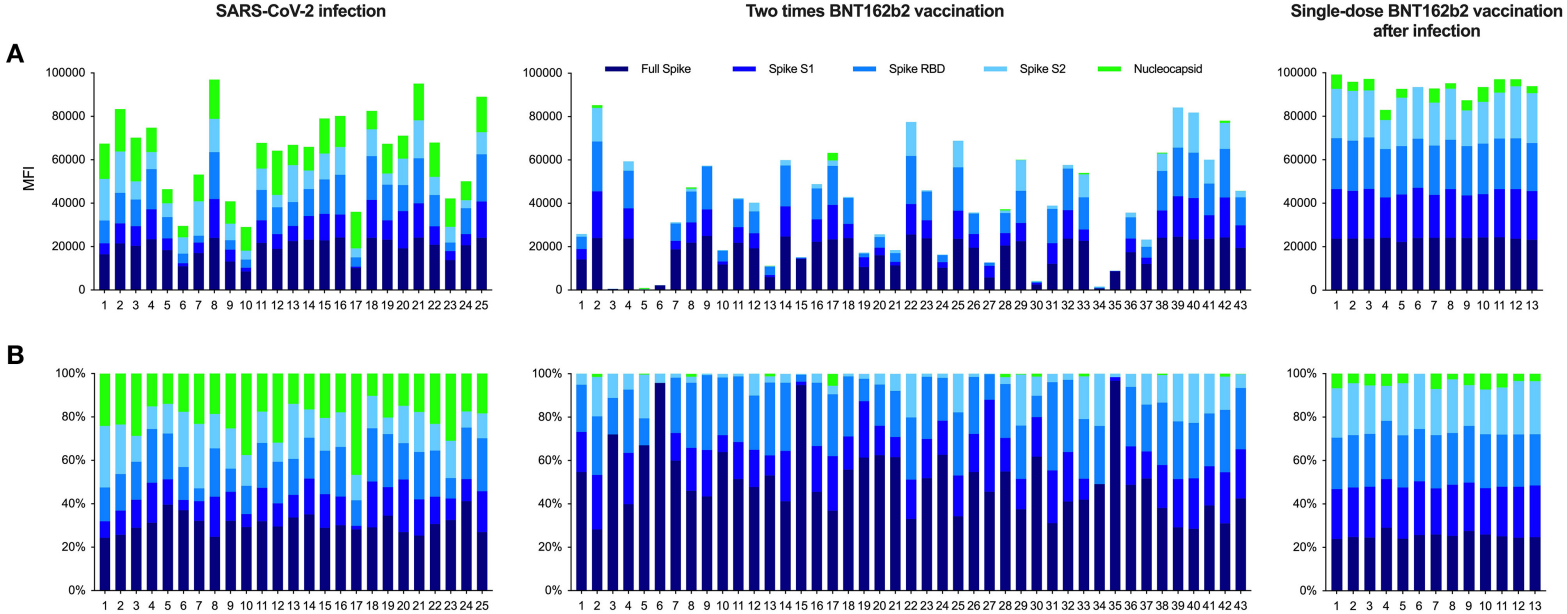
Reactivity patterns for different SARS-CoV-2 target antigens in hemodialysis patients after COVID-19 infection, complete BNT162b2 vaccination, and in previously infected patients receiving a single dose of BNT162b2. Reactivity patterns for five different SARS-CoV-2 target antigens, presented as a histogram **(A)** and as a histogram plot **(B)** for each patient. **(A)** The height of each histogram bar indicates the cumulative mean fluorescence intensity (MFI) value for detected antibodies against the full spike, the S1 spike, the receptor-binding domain of the spike, the S2 spike, and the nucleocapsid protein of SARS-CoV-2. **(B)** The histogram plot shows the proportional contribution of each target antigen to the total MFI-value and the individual variability of antibody responses in each patient.

Antibodies against the full spike protein, the S1 spike protein, and the receptor-binding domain protein were comparable between both groups with a median (IQR) MFI of 20,862 (16,750–23,315), 8,908 (5,124–13,010), and 12,301 (7,128–17,355) in infected dialysis patients and 21,797 (11,773–23,769), 7,808 (2,901–12,556), and 12,933 (3,929–18,058) in fully vaccinated dialysis patients, respectively (Figure 3A). Reactivity of S1 proteins from six other coronaviruses were also comparable between both groups (Supplementary Figure 2). However, infected dialysis patients had higher MFI-values for the spike S2 and the nucleocapsid protein with a median (IQR) of 8,553 (6,808–14,041) and 13,076 (10,452–16,591) as compared to fully vaccinated patients with 1,515 (381–4,346) and 0 (0–139) MFI-values (*P* = 0.007 and *P* < 0.001; Figure 3A). Age- and dialysis vintage-adjusted subgroup analysis confirmed these findings (Figure 3B; Supplementary Table 3). For each individual, the cumulative MFI-value for all SARS-CoV-2 proteins tested and the proportional MFI-value as a percentage of each component protein are shown in Figure 4.

The severity of COVID-19 disease courses did not correlate with results of any of the assays performed (Supplementary Figure 3).

### Antibody Response After Single-Dose BNT162b2 mRNA Vaccination in Dialysis Patients With Prior SARS-CoV-2 Infection

With a median (IQR) of 274 (151–791), dialysis patients with prior infection developed 15- to 34-fold higher anti-S1 IgG levels than age- and dialysis vintage-adjusted patients after infection or after two times vaccination (for both *P* < 0.001; Figures 2A,B; Supplementary Table 3).

The inhibition capacity of SARS-CoV-2 antibodies was also significantly higher in previously infected single-dose vaccinated patients compared to patients after infection or patients after two times vaccination, respectively (*P* = 0.002 and *P* < 0.001). Median (IQR) percent inhibition was 97.6 (97.2–98.0) in single-dose vaccinated patients with previous infection as compared to 88.0 (71.5–95.5) and 50.7 (26.4–81.0) inhibition in age- and dialysis vintage-adjusted patient after infection or two-times vaccination (Figure 2B; Supplementary Table 3).

Moreover, single-dose vaccinated patients with prior infection showed a strong and broad reactivity against all four different fragments of the SARS-CoV-2 spike protein, whereas reactivity against the nucleocapsid protein was low (Figure 4). All 13 individuals showed MFI-values above the detection cutoff for the full spike, the spike S1, the spike receptor-binding domain, and the spike S2 protein with a median (IQR) MFI of 24,017 (23,699–24,107), 22,174 (19,849–22,800), 22,736 (22,406–23,337), and 22,254 (19,490–23,179), respectively (Figure 3A). Mean fluorescence intensity-values for all four fragments were significantly higher as compared to age- and dialysis vintage-adjusted patients after infection or two-times vaccination (Figure 3B; Supplementary Table 3). Reactivity of S1 proteins against the four community coronaviruses and MERS-CoV was comparable between groups, whereas reactivity against the SARS-CoV-1 S1 protein was significantly higher in previously infected dialysis patients vaccinated with a single-dose (for both *P* < 0.001; Supplementary Figure 2). Reactivity against the nucleocapsid protein declined in single-dose vaccinated dialysis patients 6 months after SARS-CoV-2 infection (Figure 3A). The median (IQR) MFI of 4,654 (3,251–6,344) was significantly lower as compared to age- and dialysis vintage-adjusted patients 6 weeks after SARS-CoV-2 infection with a median (IQR) MFI of 13,074 (10,452–16,591) (*P* < 0.05; Figure 3B).

**Figure 5:**
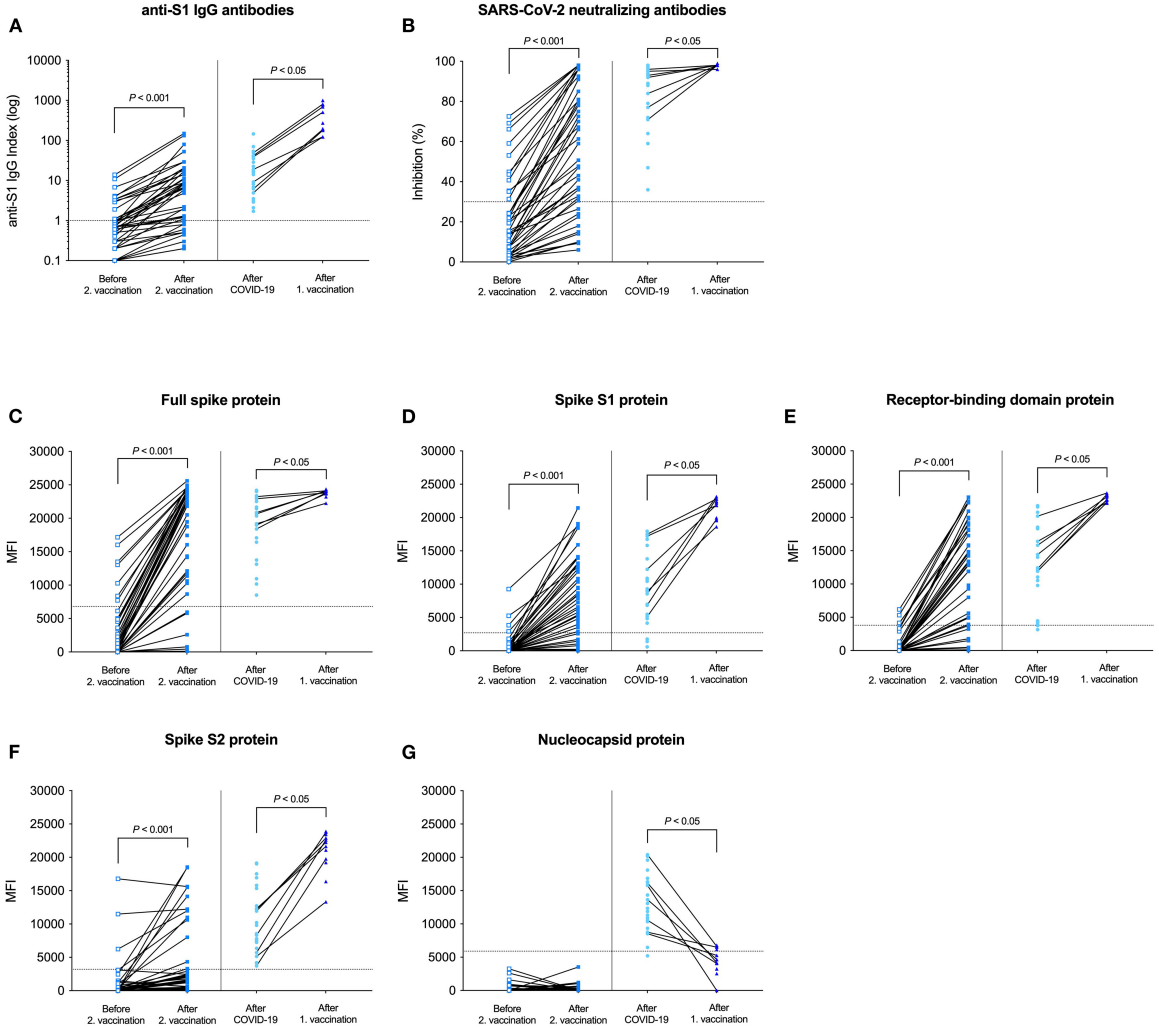
Antibody levels and antibody courses of all measurements from before second vaccination to after second vaccination and from after COVID-19 to after single-dose vaccination in patients with prior infection. SARS-CoV-2 IgG antibodies **(A)**, SARS-CoV-2 neutralizing capacity of vaccine-induced antibodies **(B)**, bead-based detection of antibodies against the full spike **(C)**, the S1 spike **(D)**, the receptor-binding domain of the spike **(E)**, the S2 spike **(F)**, and the nucleocapsid protein of SARS-CoV-2 **(G)** in hemodialysis patients before second vaccination, after second vaccination, after COVID-19 in unvaccinated patients, and after single-dose vaccination in patients with prior infection. Available individual antibody courses from before second vaccination to after second vaccination and from after COVID-19 to after single-dose vaccination in patients with prior infection are connected by a black line, respectively.

Figure 5 shows all measurements of humoral responses for all subgroups. Individual antibody courses were determined from before second vaccination to after second vaccination. In addition, antibody courses from after COVID-19 in unvaccinated patients to after single-dose vaccination in patients with prior infection were determined in patients with available sera at both time points (7 out of 13 patients). SARS-CoV-2 anti-S1 IgG, neutralizing antibody activity, and reactivity against all four different fragments of the SARS-CoV-2 spike protein significantly increased from before second vaccination to after second vaccination (for all *P* < 0.001) and from after COVID-19 in unvaccinated patients to after single-dose vaccination in patients with prior infection, respectively (for all *P* < 0.05; Figures 5A–F). In contrast, antibody levels against the nucleocapsid protein significantly decreased over time from after COVID-19 in unvaccinated patients to after single-dose vaccination in patients with prior infection (*P* < 0.05; Figure 5G).

### Local and Systemic Responses After BNT162b2 mRNA Vaccination in Dialysis Patients

Local responses were low after both the first and second BNT162b2 vaccination in dialysis patients without prior SARS-CoV-2 infection (Supplementary Figure 4). Only 2/43 (4.6%) reported mild-to-moderate pain at the injection site within 2 days after the first injection and 4/43 (9.3%) after the second injection. Systemic responses were only reported after the second dose of BNT162b2. Systemic events were fatigue 2/43 (4.6%), headache 2/43 (4.6%) and muscle ache 1/43 (2.3%).

Single-dose vaccination in previously infected dialysis patients was well-tolerated in all patients. Only 3/13 (23.1%) had pain at the injection site after single-dose vaccination and systemic reactions were not reported (Supplementary Figure 4).

## Discussion

We and others recently showed lower humoral responses to BNT162b2 mRNA vaccine in patients on hemodialysis with particularly low seroconversion rates after the first vaccine dose (3, 4). However, most COVID-19 vaccination studies were conducted in healthy individuals and the results have been directly translated to the clinical setting of dialysis patients, although cohort-adapted immunization protocols might be required (18). Since an extremely high incidence of SARS-CoV-2 infection is reported in dialysis patients from countries around the globe, vaccination strategies for previously infected patients urgently needs to be determined (19–22). Recent data suggest that single-dose rather than double-dose BNT162b2 vaccination might be reasonable for healthy individuals recovered from prior infection (12). However, since sensitivity to the first BNT162b2 mRNA vaccine dose is limited in patients on dialysis, success of single-dose vaccination in previously infected patients urgently needs to be confirmed. This is one of the first studies providing in-depth characterization of humoral responses following single-dose vaccination in dialysis patients with prior SARS-CoV-2 infection.

We investigated the antibody response to single-dose BNT162b2 vaccination in dialysis patients previously infected with SARS-CoV-2 compared with infection-naïve patients receiving standard two-dose vaccination or unvaccinated patients after COVID-19 disease. After symptomatic COVID-19 disease, dialysis patients showed higher SARS-CoV-2 specific neutralizing antibody capacity as compared to two-dose vaccinated patients without prior infection. Most importantly, single-dose vaccinated dialysis patients with prior infection had 15- to 34-fold higher anti-S1 IgG levels than age- and dialysis vintage-adjusted unvaccinated patients after infection or two-time vaccinated patients without prior infection. All 13/13 (100%) single-dose vaccinated patients with prior infection developed a broad and strong antibody response against all the different SARS-CoV-2 spike protein epitopes, accompanied by an intense neutralizing antibody capacity.

The importance of vaccination in previously infected patients stems from the decreasing SARS-CoV-2 antibody titers over time, which have been described in detail in non-dialysis patients (23). Notably, new emerging variants of concern are posing a challenge, where previously developed antibodies might not suffice to prevent re-infection. Vaccination has shown to add benefit of an increased antibody response against variants of concern such as B.1.351 or P.1 also in previously infected healthy individuals (24). These data highlight the importance of (re-)vaccination in immunocompromised patients such as patients on hemodialysis. Because of the low seroconversion rate and the attenuated neutralizing antibody titers after vaccination, this cohort is especially vulnerable for symptomatic infections with upcoming SARS-CoV-2 variants. Recent studies demonstrated that healthy individuals with previous SARS-CoV-2 infection develop strong humoral and cellular responses to a single dose of BNT162b2 (12, 25). However, these studies were limited to health care workers only and were small in sample size (12, 25). Our own and other studies on humoral responses in infection-naïve patients on hemodialysis have shown low vaccine responses, posing the imminent question whether single-dose vaccination in these patients is sufficient (26, 27). Attias et al. showed that patients receiving hemodialysis with a previous SARS-CoV-2 infection have high levels of anti-S1 IgG antibodies (28). Since older age was associated with lower seropositivity rate, confirming our own data, we performed an age- and dialysis vintage-adjusted subgroup analysis for every assay (3). We showed for the first time a broad and strong antibody response against all different SARS-CoV-2 spike protein epitopes with a high neutralizing capacity in single-dose vaccinated dialysis patients with prior infection. This is even more remarkable because we have only recently shown that a single dose of BNT162b2 mRNA vaccine is unable to exert a protective effect against SARS-CoV-2 in most infection-naïve patients on hemodialysis. Interestingly, antibodies against the nucleocapsid protein declined in single-dose vaccinated patients with prior infection whereas antibodies against the highly immunogenic receptor-binding domain that account for up to 90% of neutralizing SARS-CoV-2-specific antibodies significantly increased. The lack of vaccine-induced increase in anti-nucleocapsid antibodies in our previously infected patients represents the specificity of BNT162b2 vaccination encoding the full-length SARS-CoV-2 spike protein (29). Given the ongoing shortage of vaccine in most countries around the world, our data suggest that single-dose vaccination in previously infected dialysis patients might be reasonable. However, it remains to be investigated whether enhanced vaccine-induced antibody responses in previously infected dialysis patients will show differential longevity compared to boosted vaccines.

Data from studies published until now indicate that dialysis patients after symptomatic SARS-CoV-2 infection are able to mount a sustained antibody response (30, 31). La Milia et al. showed that 15/15 (100%) symptomatic dialysis patients developed SARS-CoV-2 spike IgG antibodies (30). Titers subsequently declined but seropositivity persisted in all patients after 6 months whereas asymptomatic dialysis patients showed low antibody responses (30). However, little is known about differences in humoral responses between twice vaccinated and SARS-CoV-2 infected dialysis patients. We showed that although anti-S1 IgG antibodies were comparable between infected and twice vaccinated dialysis patients, neutralizing antibody capacity was higher in SARS-CoV-2 infected individuals even after adjusting for age and dialysis vintage. Bead-based analysis of different SARS-CoV-2 specific antibodies revealed comparable antibodies against the receptor-biding domain of the spike protein but a more diverse antibody repertoire with reactivity against the spike S2 protein as well as the nucleocapsid protein after infection. Higher antibody titers have been reported in patients with more severe illness than those with asymptomatic disease in both healthy controls and patients on dialysis (32, 33). We found no association between the severity of COVID-19 disease and the level of IgG response or neutralizing antibodies; however, our SARS-CoV-2-infected group did not include asymptomatic individuals. In line with previous studies diabetes and male sex represented risk factors for more severe COVID-19 courses (34).

First studies compared humoral responses of dialysis patients with those of kidney transplant recipients. Rincon-Arevalo et al. showed that most dialysis patients had detectable but significantly lower antibody levels as compared to healthy controls. In contrast, kidney transplant recipients did not develop spike-specific IgG responses, with the exception of one patient who had prior undetected SARS-CoV-2 infection (35). Another study showed seroconversion in 89% of dialysis patients compared to only 18% in kidney transplant recipients after two doses of BNT162b2 (36). Because immunization seems to be more efficient in dialysis patients compared to kidney transplant recipients, they concluded that vaccination should be strongly recommended in patients awaiting a kidney transplant (36).

We did not detect breakthrough infections in any of our different groups and data about reinfection after COVID-19 or after SARS-CoV-2 vaccination is reported infrequently until now. Lumley et al. performed a prospective longitudinal cohort study of 12,541 health care workers and found no symptomatic infections and only two PCR-positive results in health care workers with previous anti-spike antibodies (7). They suggest that previous infection, resulting in antibodies to SARS-CoV-2, is associated with protection from reinfection for most people for at least 6 months (7). However, results cannot be directly transferred to dialysis patients and infection- or vaccine-induced protection from asymptomatic or symptomatic (re-)infection needs to be urgently investigated in this cohort.

Although our data conclusively demonstrate a pronounced humoral response in single-dose vaccinated dialysis patients with prior infection, they should be interpreted with caution. First, the extent to which humoral responses contribute to SARS-CoV-2 specific vaccine protection in COVID-19 is still uncertain, and second, there are no universally validated and accepted antibody thresholds that correlate with protection against severe COVID-19 courses. Further limitations of our study are the relatively small sample size and the exclusion of infected patients who died during the first 6 weeks after onset of first COVID-19 symptoms. Our preliminary data need to be confirmed by further testing including the cellular immunity, the response against other available vaccines, and most importantly the longevity of induced humoral and cellular responses.

This is one of the first studies to examine in detail the antibody response in previously infected dialysis patients after single-dose BNT162b2 mRNA vaccination. Patients with prior infection developed a broad and strong antibody reactivity against different SARS-CoV-2 spike protein epitopes with a distinct neutralizing capacity after only one vaccine dose. These promising results suggest that single-dose vaccination might also be reasonable in infected dialysis patients as previously shown in healthy controls. Further data on longevity of humoral and cellular vaccine responses are urgently needed to adapt cohort-specific immunization protocols and ultimately protect our most vulnerable cohort—patients on maintenance hemodialysis.

## Data Availability Statement

The raw data supporting the conclusions of this article will be made available by the authors, without undue reservation.

## Ethics Statement

The studies involving human participants were reviewed and approved by University of Heidelberg. The patients/participants provided their written informed consent to participate in this study.

## Author Contributions

CSp, CM, KK, and LB have contributed to planning of the study. CSp, MB, DG, CN, FK, MS, JG, MK, PR, AH, GP, PS, CSü, and LB have contributed to performing the experiments and collecting the data. CSp, CM, MS, AH, GP, PS, MZ, CSü, and LB have contributed to analysis and interpretation of data. CSp, CM, GP, PS, MZ, CSü, and LB have contributed to preparation and revision of the manuscript. All authors contributed to the article and approved the submitted version.

## Conflict of Interest

The authors declare that the research was conducted in the absence of any commercial or financial relationships that could be construed as a potential conflict of interest.

## Publisher's Note

All claims expressed in this article are solely those of the authors and do not necessarily represent those of their affiliated organizations, or those of the publisher, the editors and the reviewers. Any product that may be evaluated in this article, or claim that may be made by its manufacturer, is not guaranteed or endorsed by the publisher.
