# Supplementary material for: Humoral Responses to Single-Dose BNT162b2 mRNA Vaccination in Dialysis Patients Previously Infected With SARS-CoV-2
Source: Front Med (Lausanne). 2021 Aug 17;8:721286. doi: 10.3389/fmed.2021.721286 (PMC8415834; doi:10.3389/fmed.2021.721286)
Supplement: Supplementary file 1 [file Datas_Sheet_1.docx]

**Supplemental data**

**Table of contents:**

Supplemental Figure S1. Correlation of the SARS-CoV-2 S1 IgG index with the neutralizing antibody activity measured by a surrogate virus neutralization assay

Supplemental Figure S2. Antibodies against the S1 spike protein of the 4 community coronaviruses, MERS-CoV and SARS-CoV-1

Supplemental Figure S3. SARS-CoV-2 antibody response and neutralizing capacity in dialysis patients with different COVID-19 disease severity courses

Supplemental Figure S4. Local and systemic responses after BNT162b2 mRNA vaccination in dialysis patients

Supplemental Table S1. Baseline characteristics of age-matched groups

Supplemental Table S2. COVID-19 disease courses of age-matched dialysis patients

Supplemental Table S3. SARS-CoV-2 antibody response and neutralizing capacity of age-matched groups

Supplemental Table S4. Cutoff values of the SARS-CoV-2 specific bead-based multiplex assay

Supplemental Table S5. Definition of COVID-19 disease severity

Supplemental Methods

- Side effect questionnaire
- Plate-based SARS-CoV-2 surrogate virus neutralizing assay

**Supplemental Figure S1.** Correlation of the SARS-CoV-2 S1 IgG index with the neutralizing antibody activity measured by a surrogate virus neutralization assay

**
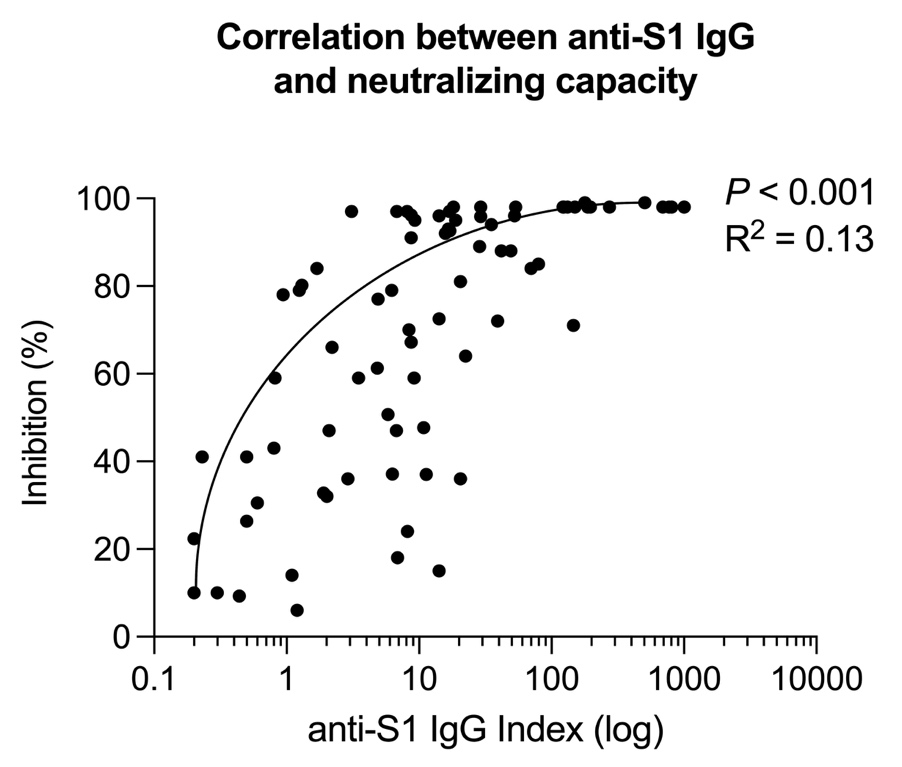
**

Correlation between the S1 antigen-specific SARS-CoV-2 IgG index and the neutralizing antibody activity measured by a surrogate virus neutralization assay in dialysis patients. SARS-CoV-2 IgG indices are represented as a semi-quantitative index. Neutralizing capacity determined by antibody-mediated inhibition of the SARS-CoV-2 receptor-binding domain:angiotensin-converting enzyme 2 interaction is expressed as a percentage.

**Supplemental Figure S2.** Antibodies against the S1 spike protein of SARS-CoV-1, MERS-CoV, and the 4 community coronaviruses

**
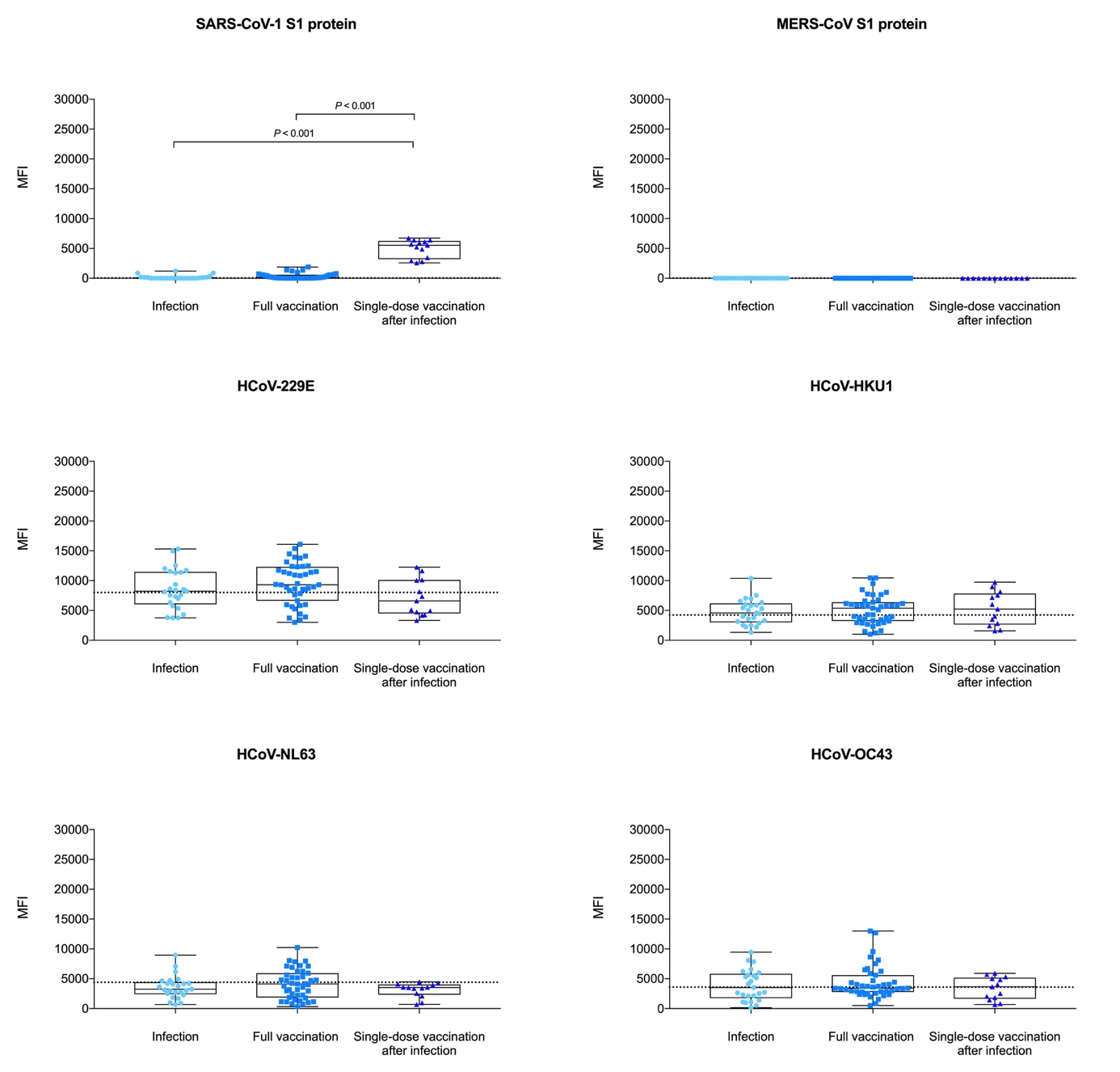
**

Detection of antibodies against SARS-CoV-1, MERS-CoV, and against the S1 spike protein of HCoV-229E, HCoV-HKU1, HCoV-NL63, and HCoV-OC43 in hemodialysis patients after COVID-19 infection, complete BNT162b2 vaccination, and in previously infected patients receiving a single dose of BNT162b2. The x-axis indicates the group of patients and the y-axis represents the mean fluorescence intensity (MFI) value of the reactivity. The dashed line represents the cutoff for detection, respectively.

**Supplemental Figure S3.** SARS-CoV-2 antibody response and neutralizing capacity in dialysis patients with different COVID-19 disease severity courses

**
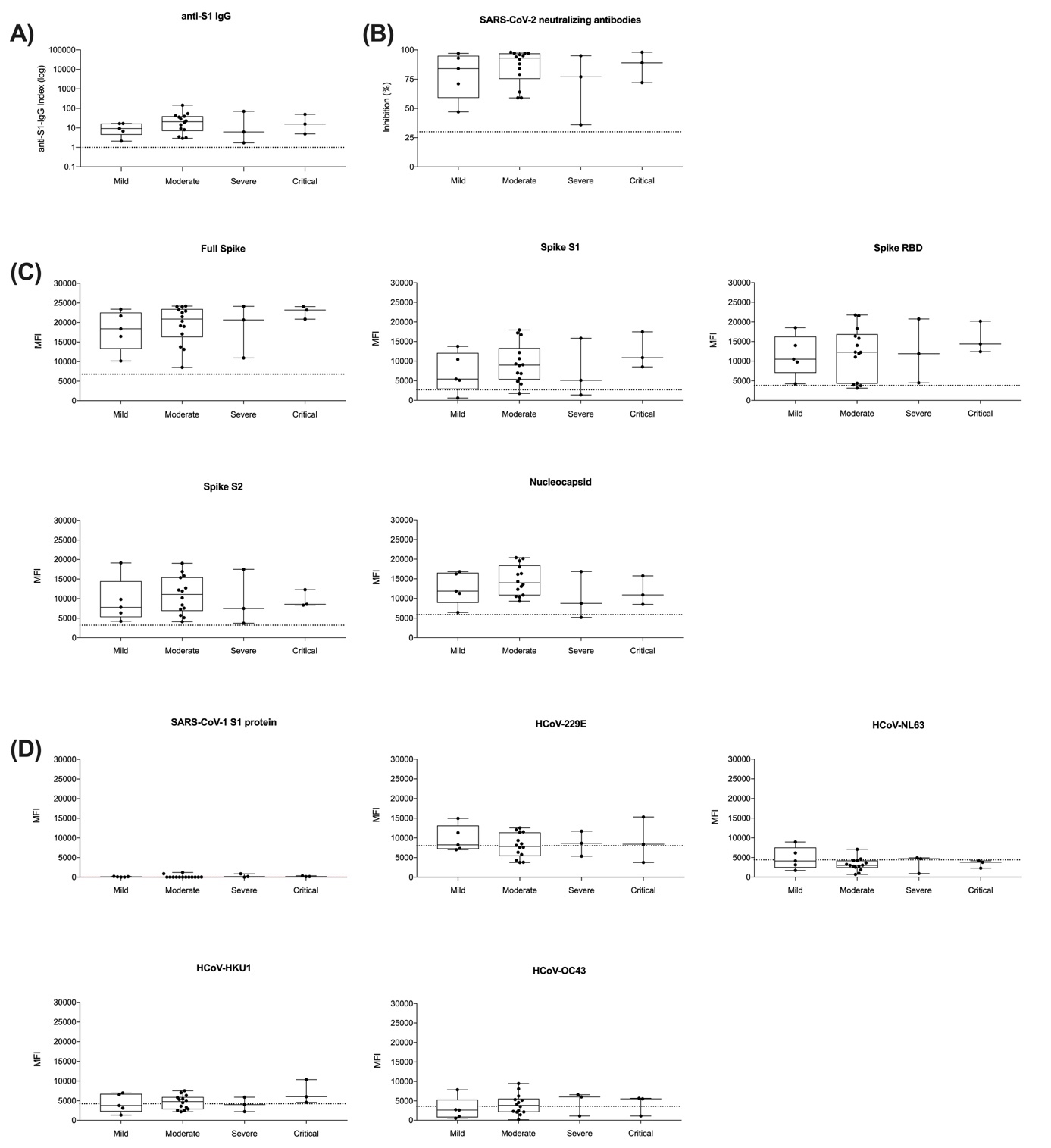
**

(A) SARS-CoV-2 IgG antibodies are represented logarithmically as an anti-S1-IgG index. The dashed line represents the cutoff for SARS-CoV-2 IgG antibody detection with a semi-quantitative index of $<$ 1 classified as negative, and a value of $\geq1$ or higher classified as positive, according to the manufacturer’s instructions. (B) SARS-CoV-2 neutralizing capacity was determined by a virus neutralization test and antibody-mediated inhibition of the SARS-CoV-2 receptor-binding domain:angiotensin-converting enzyme 2 interaction is expressed as a percentage. Binding inhibition >30% indicates presence of SARS-CoV-2 neutralizing antibodies above the limit of detection of this test. (C) For IgG antibodies against the full spike, the S1 spike, the receptor-binding domain of the spike, the S2 spike, and the nucleocapsid protein of SARS-CoV-2, the mean fluorescence intensity (MFI) is shown for each target on the y-axis. (D) For IgG antibodies against the SARS-CoV-1 S1 spike and against S1 spike proteins of different community coronaviruses, the mean fluorescence intensity (MFI) is shown for each target on the y-axis. The dashed line indicates the cutoff for detection for each target antigen. Given on the x-axis are the different COVID-19 disease severity courses, namely mild, moderate, severe, and critical.

**Supplemental Figure S4.** Local and systemic reactogenicity after BNT162b2 mRNA vaccination in dialysis patients


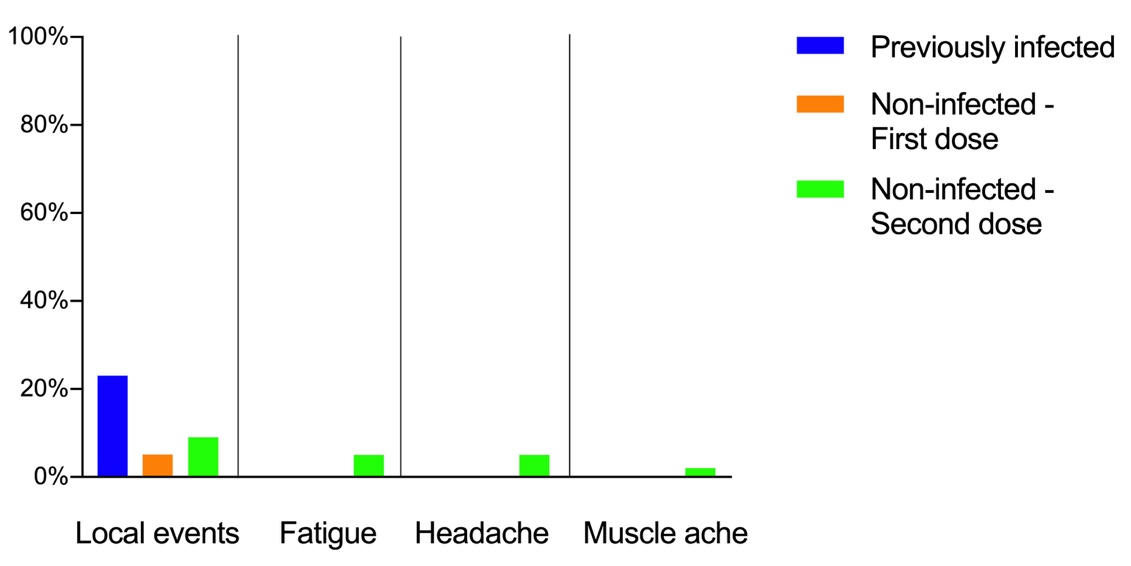


Local events and systemic events, namely fatigue, headache, and muscle ache in previously COVID-19 infected hemodialysis patients receiving a single dose of BNT162b2 and in COVID-19 naïve individuals after the first and second dose of BNT162b2, respectively. Other systemic events after vaccination such as fever, chills, vomiting, diarrhoea, joint pain, swollen lymph nodes, or the use of medication are not shown on this figure as not stated by any of the three groups.

**Supplemental Table S1.** Baseline characteristics of age-matched groups

|  | **Group 1**  **(COVID-19 infected)** | **Group 2**  **(Twice BNT162b2 vaccinated)** | **Group 3^b^**  **(Single-dose BNT162b2 vaccinated after infection)** | ***P* value** |
| --- | --- | --- | --- | --- |
| Number of patients, N | 20 | 30 | 13 |  |
| Age at enrollment (years), median (IQR) | 77 (65-83) | 81 (74-82) | 79 (74-83) | 0.65 |
| Gender, female (%) | 6 (30) | 9 (30) | 2 (15) | 0.57 |
| BMI, median (IQR) | 26 (24-29) | 25 (21-28) | 25 (23-27) | 0.34 |
| Dialysis vintage (months), median (IQR) | 36 (23-75) | 43 (23-85) | 41 (19-78) | 0.39 |
| Cause of nephropathy  Diabetes, N (%)  Vascular, N (%)  Polycystic kidney disease, N (%)  Glomerulonephritis, N (%)  Chronic pyelonephritis, N (%)  Other, N (%) | 4 (20)  6 (30)  1 (5)  4 (20)  0 (0)  5 (25) | 6 (20)  5 (17)  3 (10)  7 (21)  1 (3)  9 (30) | 2 (15)  3 (23)  0 (0)  4 (31)  0 (0)  4 (31) | 0.94  0.75  0.42  0.87  0.79  0.91 |
| Comorbidities  Arterial hypertension, N (%)  Diabetes, N (%)  Cancer, N (%)  Immunosuppressants, N (%)  Previous transplant, N (%)  Smoker (active and former), N (%)  CAD, N (%)  PAD, N (%)  Chronic lung disease, N (%)  Chronic liver disease, N (%) | 17 (80)  9 (45)  5 (25)  3 (15)  1 (5)  6 (30)  13 (65)  5 (25)  7 (28)  4 (20) | 30 (100)  11 (37)  8 (27)  6 (20)  3 (10)  10 (30)  18 (60)  11 (37)  9 (30)  3 (10) | 10 (77)  5 (39)  3 (23)  3 (23)  1 (8)  5 (39)  7 (71)  4 (31)  4 (31)  2 (15) | 0.036^a^  0.82  0.91  0.79  0.82  0.95  0.89  0.31  0.98  0.34 |

BMI, body mass index; CAD, coronary artery disease; PAD, peripheral artery disease; ^a^ statistically significant; ^b^ Group 3 contains 7 patients of group 2 after single-dose BNT162b2 vaccination

**Supplemental Table S2.** COVID-19 disease courses of age-matched dialysis patients

| **Disease characteristics** | **COVID-19 infected dialysis patients (N=20)** |
| --- | --- |
| COVID-19 disease severity^a^  Mild, N (%)  Moderate, N (%)  Severe, N (%)  Critical, N (%) | 3 (15)  12 (60)  3 (15)  2 (10) |
| Initial symptoms  Fever, N (%)  Cough, N (%)  Headache, N (%)  Diarrhea, N (%)  Fatigue, N (%)  Dyspnoe, N (%) | 16 (80)  9 (45)  1 (5)  1 (5)  10 (50)  8 (40) |
| Oxygen supply, N (%) | 9 (45) |
| Non-invasive ventilation, N (%) | 5 (25) |
| Invasive ventilation, N (%) | 2 (10) |
| Use of vasopressors, N (%) | 2 (10) |
| ICU stay, N (%) | 5 (25) |
| Immunomodulatory therapy  Dexamethasone, N (%)  Plasma exchange, N (%) | 5 (25)  3 (15) |
| Days of PCR positivity, median (IQR) | 32 (16-39) |

PCR, polymerase chain reaction; ICU, intensive care unit; ^a^ previously used grading systems for COVID-19 disease severity ^16,17^

**Supplemental Table S3.** SARS-CoV-2 antibody response and SARS-CoV-2 neutralizing capacity of age- and dialysis vintage-matched groups

|  | **Group 1**  **(COVID-19 infected)** | **Group 2**  **(Twice BNT162b2 vaccinated)** | **Group 3^b^**  **(Single-dose BNT162b2 vaccinated after infection)** | ***P* value** |
| --- | --- | --- | --- | --- |
| Number of patients, N | 20 | 30 | 13 |  |
| Neutralizing antibodies (inhibition in %), median (IQR)  Responder/Non-Responder, N/N (%) | 94 (80-97)  20/20 (100) | 56 (32-91)  24/30 (80) | 98 (97-98)  13/13 (100) | 1 vs 2; *P*=0.047^a^  1 vs 3; *P*=0.004^a^  2 vs 3; P<0.001^a^ |
| Anti-S1 IgG antibodies (index), median (IQR)  Responder/Non-Responder, N/N (%) | 18 (8-41)  20/20 (100) | 8 (1-21)  24/30 (80) | 274 (151-791)  13/13 (100) | 1 vs 2; *P*=0.31  1 vs 3; *P<*0.001^a^  2 vs 3; P<0.001^a^ |
| Full Spike antibodies (MFI),  median (IQR)  Responder/Non-Responder, N/N (%) | 21,546 (17,547-24,133)  20/20 (100) | 22,035 (13,652-25,589)  26/30 (87) | 24,017 (23,699-24,355)  20/20 (100) | 1 vs 2; *P*=0.99  1 vs 3; *P*=0.010^a^  2 vs 3; P=0.011^a^ |
| Anti-S1 antibodies (MFI),  median (IQR)  Responder/Non-Responder, N/N (%) | 9,185 (5,217-15,321)  19/20 (95) | 8,373 (4,723-19,071)  25/30 (83) | 22,174 (19,848-22,800)  13/13 (100) | 1 vs 2; *P*=0.99  1 vs 3; *P<*0.001^a^  2 vs 3; P<0.001^a^ |
| RBD antibodies (MFI),  median (IQR)  Responder/Non-Responder, N/N (%) | 12,363 (10,652-18,006)  19/20 (95) | 13,736 (5,202-22,446)  25/30 (83) | 22,736 (22,406-23,337)  13/13 (100) | 1 vs 2; *P*=0.99  1 vs 3; *P<*0.001^a^  2 vs 3; P<0.001^a^ |
| Anti-S2 antibodies (MFI),  median (IQR)  Responder/Non-Responder, N/N (%) | 10,014 (7,509-15,692)  20/20 (95) | 1,569 (482-10,715)  10/30 (33) | 22,254 (19,490-23,179)  13/13 (100) | 1 vs 2; *P*=0.012^a^  1 vs 3; *P*=0.004^a^  2 vs 3; P<0.001^a^ |
| Anti-nucleocapsid antibodies (MFI), median (IQR)  Responder/Non-Responder, N/N (%) | 12,709 (10,404-16,724)  19/20 (95) | 0 (0-14)  0/30 (0) | 4,654 (3,251-6,344)  4/13 (31) | 1 vs 2; *P<*0.001^a^  1 vs 3; *P*=0.030^a^  2 vs 3; P<0.001^a^ |
| Anti-SARS-CoV-1 S1 antibodies (MFI), median (IQR)  Responder/Non-Responder, N/N (%) | 27 (0-209)  9/20 (45) | 230 (42-599)  23/30 (77) | 5,541 (3,210-6,260)  13/13 (100) | 1 vs 2; *P*=0.22  1 vs 3; *P<*0.001^a^  2 vs 3; P<0.001^a^ |
| Anti-229E S1 antibodies (MFI), median (IQR)  Responder/Non-Responder, N/N (%) | 8,174 (5,900-11,623)  11/20 (55) | 9,051 (5,796-12,331)  17/30 (57) | 6,603 (4,514-10,105)  5/30 (17) | 1 vs 2; *P*=0.99  1 vs 3; *P*=0.65  2 vs 3; P=0.23 |
| Anti-HKU1 S1 antibodies (MFI), median (IQR)  Responder/Non-Responder, N/N (%) | 4,544 (2,999-5,988)  11/20 (55) | 5,263 (3,059-6,535)  16/30 (53) | 5,248 (2,635-7,847)  9/13 (69) | 1 vs 2; *P*=0.99  1 vs 3; *P*=0.99  2 vs 3; P=0.99 |
| Anti-NL63 S1 antibodies (MFI), median (IQR)  Responder/Non-Responder, N/N (%) | 3,154 (1,969-4,546)  5/20 (25) | 3,193 (1,709-10,225)  11/30 (37) | 3,545 (2,342-4,040)  2/13 (15) | 1 vs 2; *P*=0.99  1 vs 3; *P*=0.99  2 vs 3; P=0.99 |
| Anti-OC43 S1 antibodies (MFI), median (IQR)  Responder/Non-Responder, N/N (%) | 3,146 (1,579-5,893)  10/20 (50) | 3,770 (2,974-6,092)  17/30 (57) | 3,662 (1,656-5,183)  8/13 (62) | 1 vs 2; *P*=0.67  1 vs 3; *P*=0.99  2 vs 3; P=0.51 |

MFI; mean fluorescence intensity; ^a^ statistically significant; ^b^ Group 3 contains 7 patients of group 1 after single-dose BNT162b2 vaccination

**Supplemental Table S4.** Cutoff values of the SARS-CoV-2 specific bead-based multiplex assay

| **Target** | **Cut-off (MFI)** |
| --- | --- |
| SARS-CoV-2 Spike | 6800 |
| SARS-CoV-2 Spike S1 | 2700 |
| SARS-CoV-2 Spike RBD | 3800 |
| SARS-CoV-2 Spike S2 | 3200 |
| SARS-CoV-2 Nucleocapsid Protein | 5900 |
| HCoV-229E Spike S1 | 8012 |
| HCoV-HKU1 Spike S1 | 4235 |
| HCoV-NL63 Spike S1 | 4407 |
| HCoV-OC43 Spike S1 | 3599 |
| MERS-CoV Spike S1 | 21 |
| SARS-CoV-1 Spike S1 | 41 |

MERS, middle east acute respiratory syndrome; MFI, mean fluorescence intensity; SARS, severe acute respiratory syndrome

**Supplemental Table S5.** Definition of COVID-19 disease severity

| **COVID-19 Severity** | **Endpoint definitions** |
| --- | --- |
| *Mild* | Mild symptoms (i.e. fever, cough, change in taste of smell), no dyspnea; no need for hospital admission |
| *Moderate* | Clinical or radiographic evidence of lower respiratory tract disease; need for hospital admission |
| *Severe* | Oxygen saturation <94%, respiratory rate ≥30 breaths/min; need for non-invasive ventilation |
| *Critical* | Respiratory failure, shock, multiorgan dysfunction or failure; need for invasive ventilation |

**Supplemental Methods**

Side effect questionnaire

1. **Side effects after 1st vaccination:**

⃝ yes ⃝ no

1. **If yes, please mark the appropriate side effects you had:**

⃝ local events (such as pain at injection site, redness, swelling)

⃝ fever ⃝ fatigue ⃝ headache ⃝ chills

⃝ vomiting ⃝ diarrhea ⃝ muscle pain ⃝ joint pain

⃝ swollen lymph nodes ⃝ others, such as ___________________

1. **In response to above mentioned side-effects, I took the following medication**

⃝ NSAID (i.e. Ibuprofen)

⃝ Paracetamol

⃝ Metamizol (i.e. Novalgin)

⃝ Aspirin

⃝ others, such as ___________________

1. **Side effects after 2nd vaccination:**

⃝ yes ⃝ no

1. **If yes, please mark the appropriate side effects you had:**

⃝ local events (such as pain at injection site, redness, swelling)

⃝ fever ⃝ fatigue ⃝ headache ⃝ chills

⃝ vomiting ⃝ diarrhea ⃝ muscle pain ⃝ joint pain

⃝ swollen lymph nodes ⃝ others, such as ___________________

1. **In response to above mentioned side-effects, I took the following medication**

⃝ NSAID (i.e. Ibuprofen)

⃝ Paracetamol

⃝ Metamizol (i.e. Novalgin)

⃝ Aspirin

⃝ others, such as ___________________

Plate-based SARS-CoV-2 surrogate virus neutralizing assay

This plate-based SARS-CoV-2 surrogate virus neutralizing assay mimics the virus-host interaction by direct protein-protein interaction using purified receptor-binding domain protein from the viral spike protein and the host cell receptor angiotensin converting enzyme 2 (ACE2). The antibodies in serum samples were incubated with SARS-CoV-2 receptor-binding domain horse-radish peroxidase and added to ACE2 coated wells. The reactions were developed using 3,3’,5,5’-tetramethylbenzidine as substrate.

Optical density at 450 nm was measured in each well and the percent (%) inhibition was calculated as follows:

$$\mathbf{Inhibition=}\left( \mathbf{1-}\left( \frac{\mathbf{OD value of Sample}}{\mathbf{OD value of Negative Control}} \right) \right)\boldsymbol{X 100\%}$$
